# Genomic insights into the probiotic potential of *Bacillus velezensis* D-18 for aquaculture

**DOI:** 10.3389/fmicb.2026.1824441

**Published:** 2026-05-18

**Authors:** Luis Monzón-Atienza, Álvaro Lorenzo Felipe, Nicolás Cabrera-Guerlé, Antonio Gómez-Mercader, Camila Carlino-Costa, José Ramos-Vivas, Meiling Zhang, Daniel Montero, Félix Acosta, Jorge Galindo-Villegas

**Affiliations:** 1 Grupo de Investigación en Acuicultura (GIA), IU-ECOAQUA, Universidad de Las Palmas de Gran Canaria, Las Palmas, Spain; 2 Department of Genomics, Faculty of Biosciences and Aquaculture, Nord University, Bodø, Norway; 3 Research Group Biotechnology of Nutraceuticals and Bioactive Compounds (BIONUC), Area of Microbiology, Department of Functional Biology, Universidad de Oviedo, Oviedo, Spain; 4 Laboratory of Aquaculture Nutrition and Environmental Health (LANEH), School of Life Sciences, East China Normal University, Shanghai, China

**Keywords:** antimicrobial metabolite biosynthesis, biosynthetic gene clusters, comparative genomics, host-microbe interactions, mucosal adhesion, quorum-quenching

## Abstract

The identification of safe and functionally robust probiotics is a key challenge for sustainable aquaculture and requires genome-based validation of candidate strains. Here, we performed an integrative genomic characterization of *Bacillus velezensis* D-18, a strain previously shown to enhance disease resistance in European seabass. Whole-genome sequencing generated a draft genome of approximately 4.06 Mb containing 4,178 protein-coding genes and 84 RNA genes. Comparative genomics confirmed the taxonomic placement of the strain within the *B. velezensis* lineage and its clear separation from pathogenic members of the *Bacillus cereus* group. Genome-wide screening detected no acquired antimicrobial resistance genes or virulence determinants, supporting the biosafety of the strain. Functional annotation revealed genetic determinants associated with quorum quenching, biofilm formation, stress tolerance, and antimicrobial activity. AntiSMASH analysis further identified seven biosynthetic gene clusters encoding bioactive metabolites including surfactin, fengycin, bacilysin, and macrolactin. In addition, molecular docking analyses suggested potential interactions between bacterial proteins and mucin glycoproteins, consistent with previously reported mucus adhesion. Together, these findings provide a genomic framework supporting the probiotic potential of *B. velezensis* D-18 for aquaculture applications.

## 1 Introduction

The rapid intensification of marine aquaculture over the past decade has markedly increased the incidence and impact of infectious diseases, particularly in high value species such as European seabass (*Dicentrarchus labrax*) (Galindo-Villegas and Mulero, 2014; Mitropoulou et al., 2026). Bacterial pathogens, notably *Vibrio* spp., remain a major constraint on production efficiency, animal welfare, and environmental sustainability (Acosta et al., 2021). Historically, disease control in aquaculture has relied heavily on the prophylactic or therapeutic use of antimicrobials (Schar et al., 2020). However, growing concerns regarding antimicrobial resistance, environmental dissemination of antibiotics, and increasingly restrictive regulatory frameworks have accelerated the search for alternative, biologically based disease-control strategies (Tao et al., 2024; Kumar et al., 2026). In this context, probiotics have emerged as promising, environmentally sustainable tools capable of enhancing host health while mitigating pathogen pressure (Montalban-Arques et al., 2015; Li et al., 2026).

In aquaculture, probiotics are now recognized not merely as growth promoters but as functional modulators of the host-microbe interface, particularly at mucosal barriers where microbial colonization, immune surveillance, and pathogen exclusion converge (Rahayu et al., 2024). These surfaces constitute critical ecological niches where microbial colonization, immune surveillance, and pathogen exclusion converge. Consequently, effective probiotic candidates must combine ecological fitness, host compatibility, and a robust safety profile, together with functional traits enabling mucosal persistence, competitive exclusion of pathogens, and modulation of host immune responses. Recent advances in whole-genome sequencing and bioinformatics provide unprecedented opportunities to dissect these traits at high resolution, facilitating a transition from empirically selected strains toward rational, genome-informed probiotic development (Aziz et al., 2024).

Members of the genus *Bacillus* are among the most extensively applied probiotics in animal production systems, including aquaculture (Cutting, 2011; Nayak, 2020). Their widespread use is largely attributable to their ability to form endospores, tolerate harsh environmental conditions, and synthesize a diverse repertoire of bioactive compounds. Several *Bacillus* species, including *B. subtilis*, *B. amyloliquefaciens*, and *B. velezensis*, have demonstrated beneficial effects on growth performance, immune competence, and disease resistance in fish (Yu et al., 2025; Shija et al., 2025; Monzón-Atienza et al., 2022). Nevertheless, the genus also encompasses opportunistic or pathogenic species, such as *B. cereus, B. anthracis*, and *B. thuringiensis*, highlighting the need for rigorous strain-level genomic safety assessment prior to probiotic deployment (Ehling-Schulz et al., 2019).

Within the *Bacillus* species complex, *Bacillus velezensis* was described as a distinct species in 2005 by Ruiz-García et al., with the type strain CR-502^T^ collected from the Velez River (Spain) during a screening for surfactant-producing bacteria (Torres et al., 2026). Since its formal classification, *B. velezensis* has emerged as a particularly promising probiotic candidate, largely due to its exceptional capacity for secondary metabolite biosynthesis, including a wide range of antimicrobial compounds, enzymes, and lipopeptides (Wang et al., 2025), together with its ability to interfere with quorum sensing interference and to form structured biofilms. These traits are increasingly recognized as key ecological determinants of microbial competitiveness and pathogen suppression in complex environments. In aquaculture systems, such properties are especially relevant, as they enable probiotics to attenuate pathogen virulence, disrupt biofilm formation, and stabilize beneficial microbial communities without exerting broad-spectrum antimicrobial pressure (Rahayu et al., 2024).

*Bacillus velezensis* D-18 was originally isolated from an aquaculture-associated environment (Monzón-Atienza et al., 2021) and has previously demonstrated strong probiotic potential in European seabass (Monzón-Atienza et al., 2022). Experimental studies have shown its capacity to adhere to fish mucus, tolerate gastrointestinal conditions, inhibit pathogenic Vibrio biofilm formation, and enhance host survival following bacterial challenge (Monzón-Atienza et al., 2024). While these phenotypic observations strongly support its functional relevance, phenotypic assays alone are insufficient to ensure strain-level safety, functional predictability, and regulatory suitability. The genomic architecture underpinning these probiotic traits, together with the absence of virulence factors and transferable antimicrobial resistance determinants, has not yet been comprehensively resolved. Moreover, from a regulatory and translational perspective, genome-resolved characterization is increasingly regarded as a prerequisite for the safe, predictable, and large-scale deployment of live microbial products in food-producing aquaculture systems (Kuebutornye et al., 2019; Rahayu et al., 2024).

Recent studies on probiotic *Bacillus* spp. have demonstrated that functional traits such as gastrointestinal tolerance, mucosal adhesion, and environmental stress resistance are underpinned by specific genomic determinants, including ATP synthase complexes, bile transporters, stress response proteins, and adhesion-related factors (Ghosh, 2025). Comparative analyses in strains such as *B. clausii* further highlight the importance of distinguish between spore and vegetative states, with spores exhibiting enhanced resilience and colonization potential under gastrointestinal conditions (Ahire et al., 2021). These findings support the use of genome-based approaches to predict probiotic functionality and safety in candidate strains.

In the present study, we therefore provide an integrative whole-genome characterization of *B. velezensis* D-18 using high-coverage genome sequencing, comparative genomics, and targeted bioinformatic analyses. Specifically, we aimed to (i) confirm the taxonomic placement and genomic integrity of the strain; (ii) assess its safety by screening for virulence- and antimicrobial resistance-associated genes; (iii) identify genetic determinants linked to probiotic-relevant functions, including mucosal colonization, quorum quenching, stress tolerance, and immune modulation; and (iv) characterize biosynthetic gene clusters (BGCs) encoding secondary metabolites with relevance for pathogen control in aquaculture. By linking genomic features to previously validated functional traits in European seabass, this study positions *B. velezensis* D-18 as a genomically validated probiotic candidate aligned with sustainable and antibiotic-free aquaculture practices.

## 2 Materials and methods

### 2.1 Isolation and characterization of *Bacillus velezensis* D-18

*Bacillus velezensis* D-18 was isolated from wastewater samples collected at a turbot aquaculture facility by the Instituto Español de Oceanografía (IEO), Santander, Spain. Following isolation, the strain was subjected to extensive *in vitro* and *in vivo* characterization at IU-ECOAQUA, University of Las Palmas de Gran Canaria (Spain), as previously described (Monzón-Atienza et al., 2021). These analyses demonstrated broad antimicrobial activity against fish pathogens, strong adhesion capacity to European seabass (*D. labrax*) skin and intestinal mucus, tolerance to gastrointestinal pH and bile conditions, absence of adverse effects on the host, and a significant increase in host survival following experimental challenge with *Vibrio anguillarum* 507. Based on these properties, the strain D-18 was selected for comprehensive genomic characterization to elucidate the genetic basis of its probiotic traits and evaluate its biosafety at the strain level.

### 2.2 DNA extraction, sequencing, assembly and annotation

Genomic DNA from *B. velezensis* D-18 was extracted using the E. Z. N. A.® Bacterial DNA Kit (Omega, Bio-tek, United States) according to the manufacturer’s instructions. DNA quality and concentration were assessed prior to sequencing. Whole-genome sequencing was performed by Macrogen (Seoul, South Korea) using Illumina paired-end technology. Sequencing libraries were prepared using the TruSeq DNA PCR-Free kit. Raw sequencing reads were evaluated for quality using FastQC (V0.11.8). Adapter sequences and low-quality reads were removed using Trimmomatic (v0.38) (Bolger et al., 2014). High-quality reads were mapped against the reference genome *B. velezensis* CBMB205 (GCF_002117165.1) using BWA (v0.7.17) (Li and Durbin, 2010), and duplicate reads were removed with Sambamba (v0.6.8) (Tarasov et al., 2015). Single nucleotide polymorphisms and other sequence variants were identified and functionally annotated using SnpEff (v4.3t) (Cingolani et al., 2012). A circular genome map was generated using the Proksee platform^1^ based on the annotated genome sequence. In parallel, filtered reads were assembled *de novo* using the Read Assembly and Annotation Pipeline Tool (RAPT). Genome annotation, including prediction of protein-coding sequences and RNA genes, was performed using the RAST server (Aziz et al., 2008). Assembly statistics, including genome size, GC content, N50, and contig number, were calculated from the final assembly.

### 2.3 Comparative genomics and safety assessment

To confirm taxonomic placement and assess genomic relatedness, Average Nucleotide Identity (ANI) analysis was conducted using JSpeciesWS^2^ (Richter et al., 2016). The genome of *B. velezensis* D-18 was compared with 19 publicly available *B. velezensis* genomes, retrieved from the JSpeciesWS (see Footnote 2) (Richter et al., 2016) database, along with the reference genome CBMB205. For contextual comparison, reference genomes of *B. amyloliquefaciens*, *B. licheniformis*, *B. subtilis*, and *Lactobacillus plantarum* were included as representative probiotic species (Amit et al., 2022; Ramirez-Olea et al., 2022; Woldemariamyohannes et al., 2020; Garvey et al., 2022). In contrast, *B. anthracis*, *B. cereus*, *B. pumilus*, and *B. thuringiensis,* including strains previously reported as pathogenic, were selected as pathogenic comparators. ANI similarity matrices were visualized as heatmaps using ClustVis^3^ (Metsalu and Vilo, 2015). Genomic biosafety evaluation was conducted using tools available at the Center for Genomic Epidemiology (CGE).^4^ Acquired antimicrobial resistance genes were identified using ResFinder 4.1,^5^ applying a minimum sequence identity threshold of 90% and a minimum coverage of 60.0% (Bortolaia et al., 2020; Florensa et al., 2022). Prediction of potential human pathogenicity was performed using PathogenFinder v1.1^6^ (Cosentino et al., 2013), based on the predicted proteome derived from the annotated genome.

### 2.4 Prediction of probiotic-associated genes and secondary metabolites

Putative probiotic-associated genes were identified by BLASTp (Protein Basic Local Alignment Tool) comparison of the *B. velezensis* D-18 genome against the NCBI non-redundant (nr) protein database. Candidate genes were selected based on sequence similarity to previously characterized proteins and subsequently curated according to functional annotations related to stress tolerance, adhesion, mucosal persistence, quorum quenching, biofilm formation, immune modulation, and antimicrobial activity, as well as literature-supported associations with probiotic traits in *Bacillus* and related Gram-positive bacteria. Identified genes were subsequently categorized according to their predicted biological functions. Secondary metabolites Biosynthetic Gene Clusters (BGCs) were predicted using the AntiSMASH platform (v7.0)^7^ (Blin et al., 2023). Detected clusters were classified according to biosynthetic type, genomic location, and similarity to known reference clusters, enabling the identification of non-ribosomal peptides, polyketides, lipopeptides, and other bioactive compounds associated with probiotic activity and pathogen suppression.

### 2.5 Molecular docking analysis of protein-mucin interactions

Protein–ligand docking simulations were performed as a hypothesis-generating approach to explore potential interactions between selected *B. velezensis* D-18 proteins and host mucin glycoproteins. Candidate bacterial proteins were selected based on predicted extracellular localization and functional annotation related to adhesion or host interaction from genome annotation. Protein structures were predicted using AlphaFold2 and prepared for docking by removal of non-protein components and addition of polar hydrogens using AutoDockTools.^8^ A representative mucin glycoprotein domain structure was retrieved from the Protein Data Bank (PDB) and used as a model receptor. Docking simulations were performed using AutoDock Vina with default parameters (Trott and Olson, 2010), and binding affinities were calculated based on predicted free energy scores. The best-scoring conformations based on predicted binding affinity (kcal·mol^−1^) were visualized using PyMOL. Docking results were interpreted qualitatively to identify potential interaction interfaces, including hydrogen bonding and hydrophobic contacts generating potential interaction patterns rather than quantitative binding predictions.

## 3 Results

### 3.1 General genome features and annotation of *Bacillus velezensis* D-18

**Figure 1:**
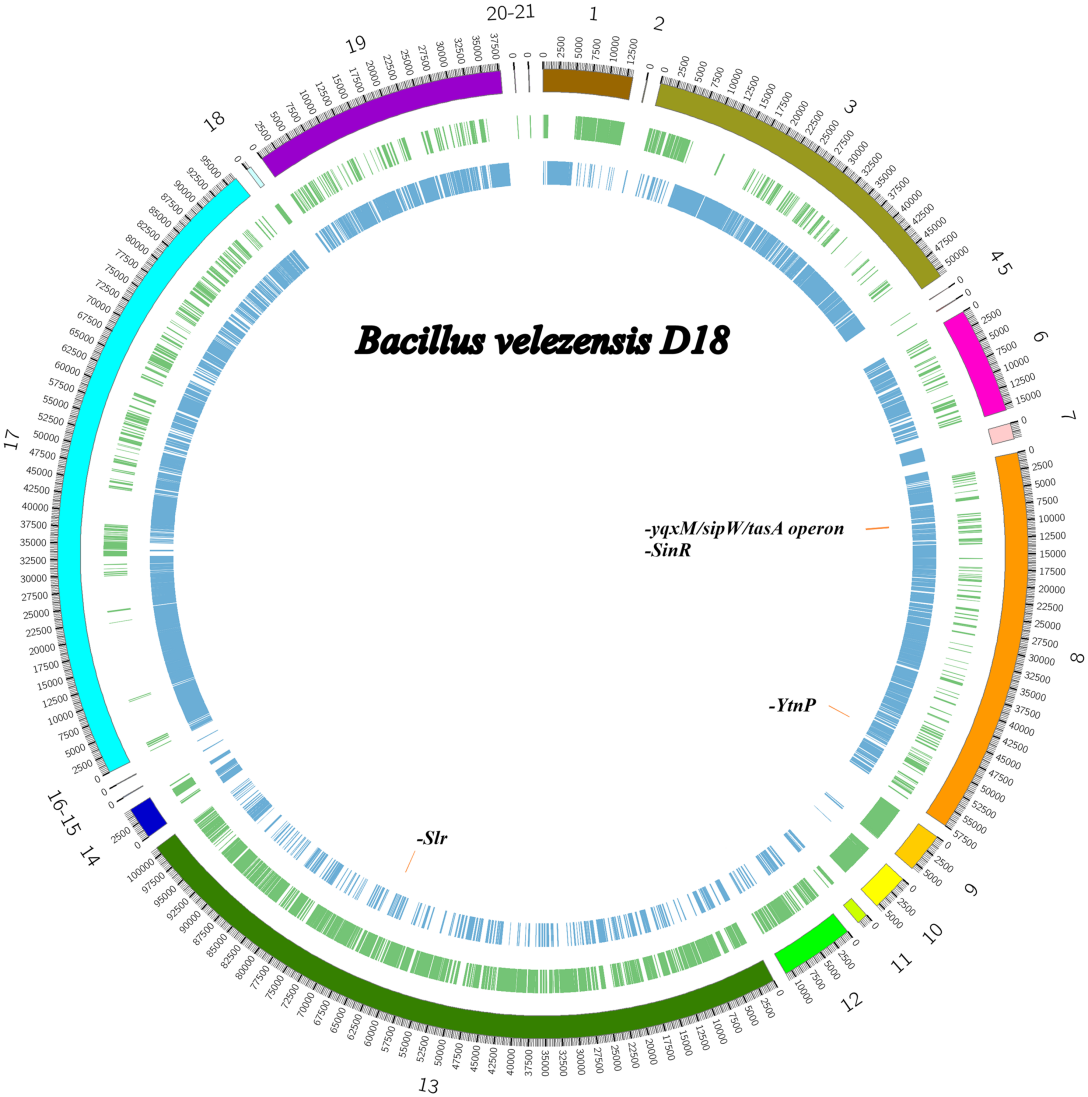
Circular representation of the *B. velezensis* D18 draft genome. The draft genome is displayed as a circular map based on the assembled contigs. The outermost ring indicates genome coordinates (in base pairs). Colored segments labeled 1–21 represent predicted biosynthetic gene clusters (BGCs) or genomic islands, with each cluster depicted in a distinct color. The inner rings show annotated coding sequences (CDSs) on the forward and reverse strands, respectively. Key functional genes and operons are labeled, including *SinR*, *SlrA*, *YtnP*, and the *yqxM–sipW–tasA* operon, associated with quorum quenching, biofilm formation, and secondary metabolism. This genomic atlas highlights loci of interest for functional and comparative genomic analyses. The map was generated using Proksee with default parameters.

Whole-genome sequencing of *B. velezensis* D-18 generated approximately 18.2 million paired-end reads, of which 97.06 and 91.85% exceeded the Q20 and Q30 quality thresholds, respectively. The draft genome spans approximately 4.06 Mb, showing 94.6% coverage relative to the reference genome *B. velezensis* CBMB205 and a mean sequencing depth of 511 X. After quality filtering and adapter trimming, approximately 17.5 million high-quality reads were retained for assembly, resulting in 21 contigs with a total length of 4,059,220 bp. The assembly exhibited an N50 of 575,178 bp and L50 of 3, with an overall G + C content of 46.60%. The overall genome structure and contig distribution are shown in Figure 1. The circular genome map further highlights the genomic localization of predicted BGCs, and key loci associated with quorum quenching and biofilm formation.

**Figure 2:**
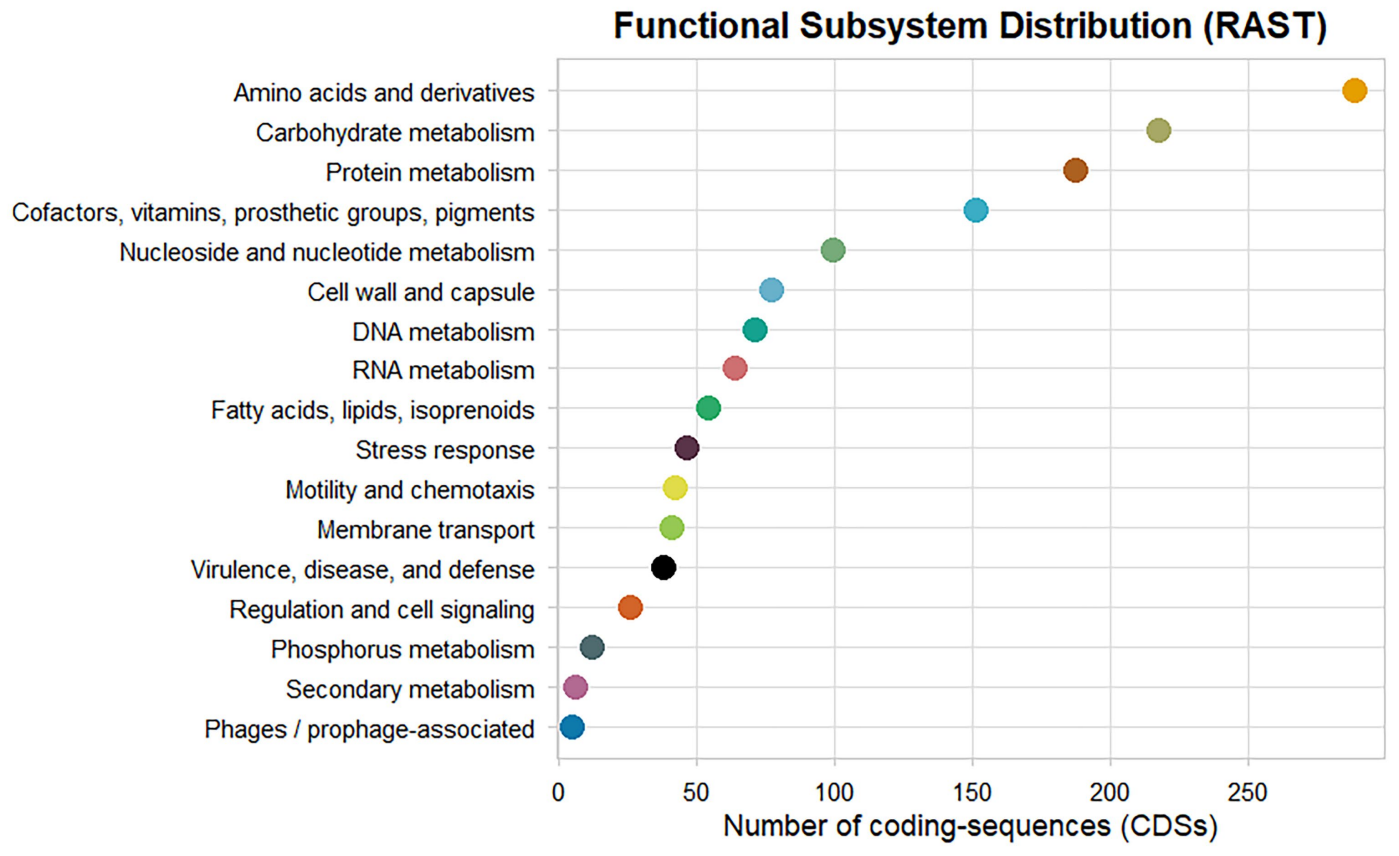
Functional distribution of RAST subsystems for the 4,178 predicted protein-coding genes of *B. velezensis* D-18. The figure highlights the relative contribution of major metabolic, cellular, and adaptive processes.

A total of 4,178 protein-coding sequences and 84 RNA-encoding genes were predicted (Supplementary Table S1), using the RAST server (Aziz et al., 2008). Functional annotation assigned these genes to 324 RAST subsystems, indicating extensive metabolic capacity and functional versatility. The most represented categories included amino acids and derivatives metabolism (288 features), carbohydrate metabolism (217 features), and protein metabolism (187 features). Additional subsystems were associated with cofactors, vitamins, prosthetic groups, and pigments (151 features), nucleoside and nucleotide metabolism (99 features), cell wall and capsule synthesis (77 features), DNA metabolism (71 features), and RNA metabolism (64 features). Genes involved in fatty acid and lipid metabolism (54 features), stress response (46 features), motility and chemotaxis (42 features), membrane transport (41 features), and virulence, disease, and defense (38 features) were also identified. Fewer features were assigned to regulation and cell signaling (26), phosphorus metabolism (12), secondary metabolism (6), and phage-associated functions (5) (Figure 2). These subsystem-based annotations derived from RAST reflect broad functional categories and are not directly equivalent to the BGCs predicted by AntiSMASH, which are described separately below.

### 3.2 Genomic determinants associated with quorum quenching and biofilm formation

**Figure 3:**
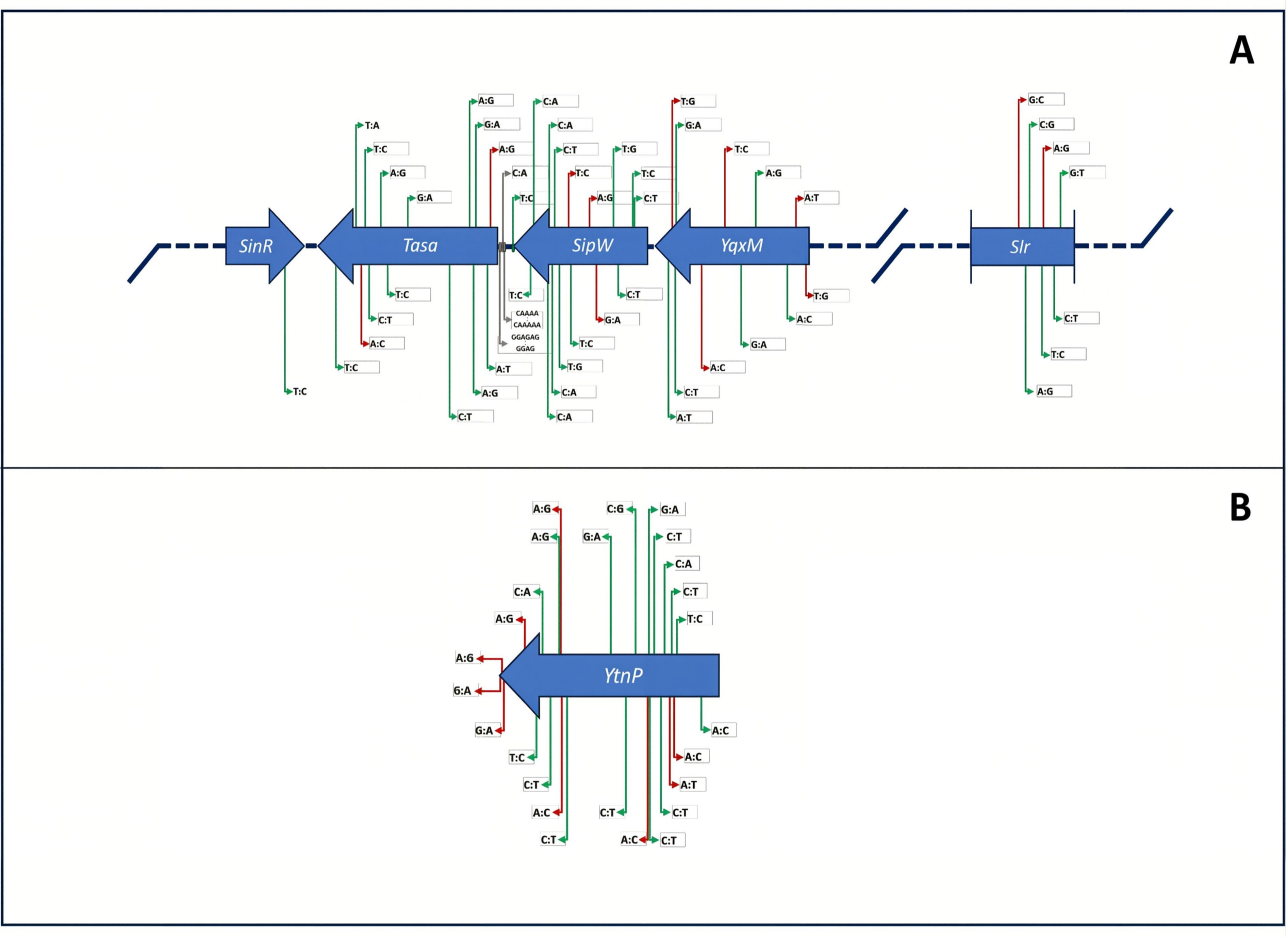
Genomic organization and sequence variation of genes associated with biofilm formation and quorum quenching in *B. velezensis* D-18. **(A)** Organization of the *yqxM–sipW–tasA* operon and its regulatory elements, including *SinR* and *SlrA*, involved in biofilm matrix production. **(B)** Genomic context of the quorum-quenching lactonase *YtnP*. Synonymous, missense, and intergenic variants relative to the reference strain CBMB205 are indicated. These loci correspond to genetic determinants previously associated with quorum quenching and biofilm formation in *Bacillus* species.

The genome of *B. velezensis* D-18 encodes the quorum-quenching lactonase gene *YtnP*, a key enzyme involved in the degradation of acyl-homoserine lactone signaling molecules, as well as the biofilm matrix operon *yqxM–sipW–tasA*, which is essential for the extracellular synthesis and assembly of the biofilm matrix. This operon is transcriptionally regulated by SinR, a negative regulator of biofilm formation, and its antagonist SlrA, which promotes matrix gene expression (Figure 3A).

The *YtnP* gene was identified as a standalone locus associated with quorum quenching activity (Figure 3B). Comparative analysis with the reference strain CBMB205 revealed sequence variation distributed across both the *YtnP* coding region and adjacent genomic regions, including putative regulatory sequences. These variations consisted predominantly of missense mutations, together with a limited number of synonymous substitutions and intergenic variants. No direct functional inference is made from these sequence variations in the absence of experimental validation.

### 3.3 Safety assessment and extended genome comparison of *Bacillus velezensis* D-18

**Figure 4:**
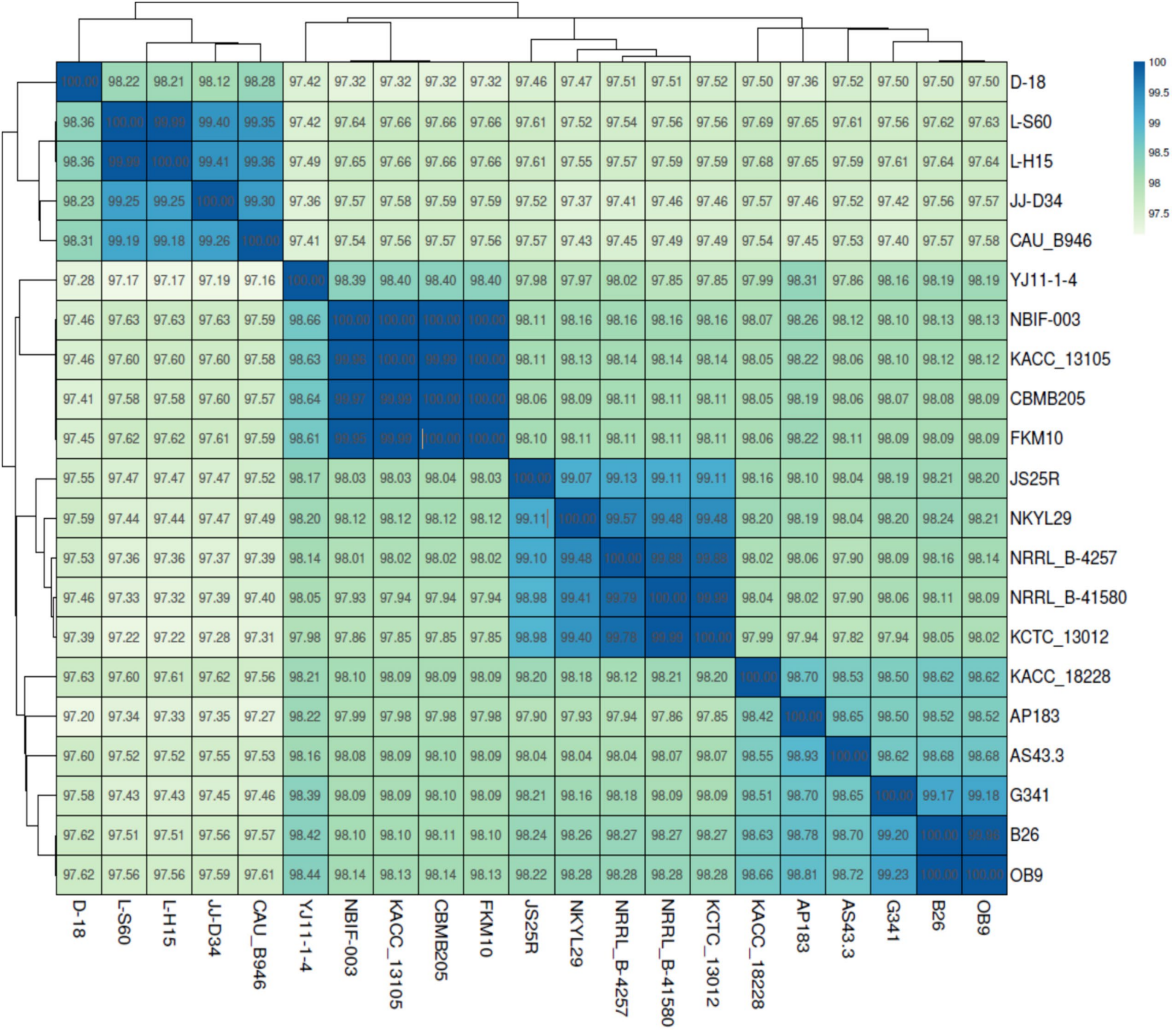
Average nucleotide identity (ANI) heatmap and hierarchical clustering of *B. velezensis* D-18 and 20 reference *B. velezensis* genomes. ANI values (percentage identity) are shown within each cell, and clustering illustrates the close genomic relatedness of strain D-18 to other members of the *B. velezensis* clade, with all comparisons exceeding the accepted species-level threshold.

**Figure 5:**
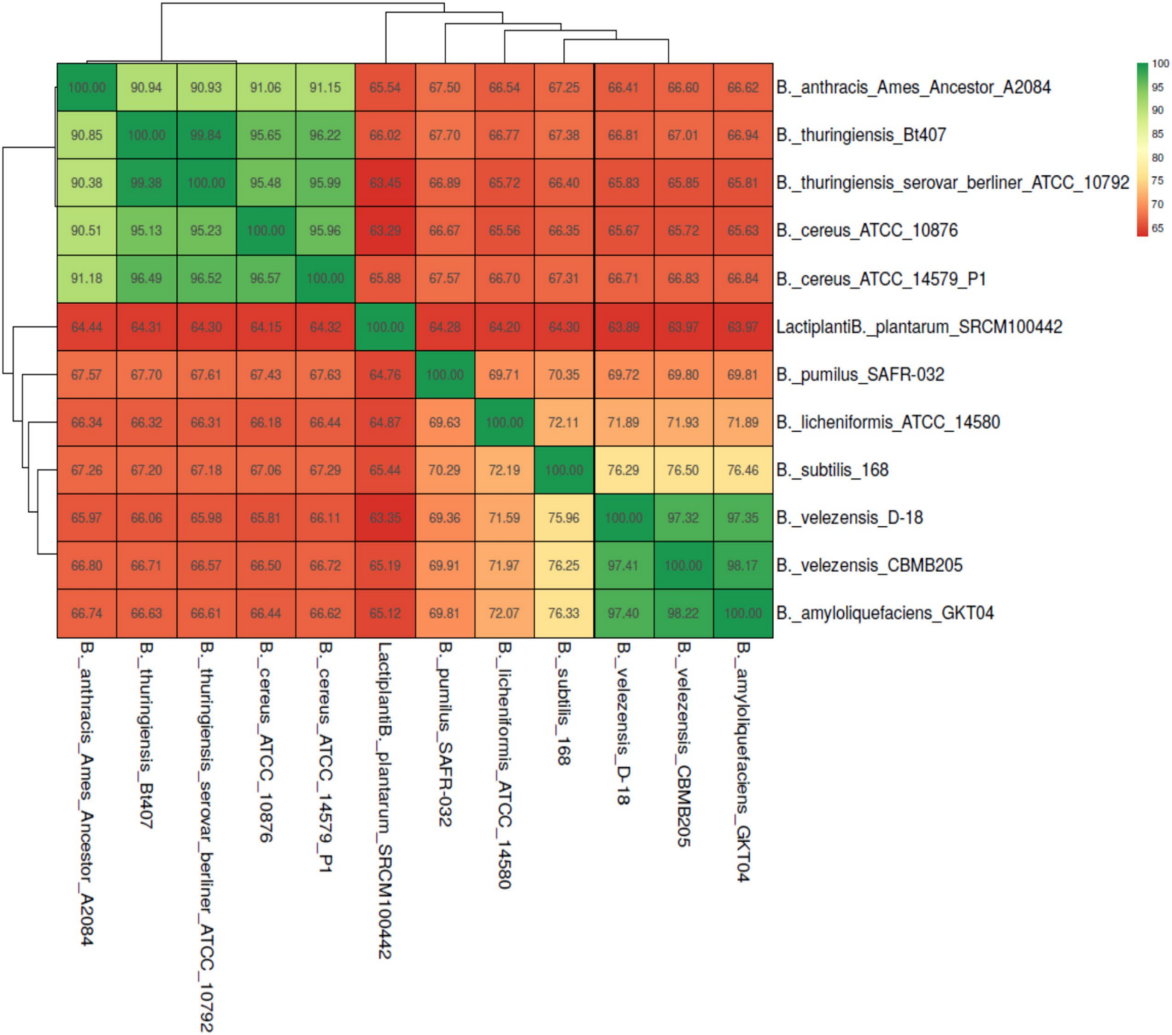
Average nucleotide identity (ANI) heatmap comparing *B. velezensis* D-18 with representative probiotic and pathogenic *Bacillus* species. Strain D-18 clusters with *B. velezensis* and *B. amyloliquefaciens*, while pathogenic species (*B. anthracis*, *B. cereus*, and *B. thuringiensis*) form a distinct, separate cluster, indicating clear genomic separation from pathogenic lineages.

Average Nucleotide Identity (ANI) analysis demonstrated that strain D-18 clusters tightly with other *B. velezensis* genomes, displaying ANI values ranging from 97.2 to 98.36%, well above the accepted species-level threshold (Figure 4). These results unequivocally confirm the taxonomic placement of strain D-18 within the *B. velezensis* clade. In contrast, interspecies ANI comparisons revealed that strain D-18 shares high genomic similarity exclusively with members of the *B. velezensis*-*B. amyloliquefaciens* group, while displaying markedly lower ANI values with other *Bacillus* species, regardless of their classification as probiotic or pathogenic (Figure 5). Notably, pathogenic species, including *B. anthracis*, *B. cereus*, and *B. thuringiensis* formed a distinct and highly conserved genomic cluster clearly separated from strain D-18.

PathogenFinder analysis was employed to evaluate the potential for human pathogenicity across representative pathogenic and non-pathogenic *Bacillus* species, with *Lactiplantibacillus plantarum* included as a probiotic reference (Table 1). Well-established pathogenic species, including *Bacillus anthracis*, *B. cereus*, and *B. thuringiensis*, exhibited high pathogenicity probability scores ranging from 0.824 to 0.874 and matched substantial numbers of pathogenic protein families (54–768). In contrast, probiotic reference species such as *B. amyloliquefaciens* GKT04, *B. subtilis* 168, *B. pumilus* SAFR-032, and *L. plantarum* SRCM100442 displayed low pathogenicity probability scores (0.184–0.240) and no matched pathogenic families. An exception was observed for *Bacillus licheniformis* ATCC 14580, which showed a high pathogenicity probability score (0.808) and matched 441 pathogenic families. Consistent with these benchmark results, all analyzed *B. velezensis* strains, including D-18, were predicted to be non-human pathogens (Table 2). Pathogenicity probability scores for *B. velezensis* ranged from 0.224 to 0.236, and none of the strains matched any pathogenic protein families. Strain D-18 exhibited a probability score of 0.227 and matched 58 non-pathogenic families, falling well within the narrow and conserved range observed across the species. Collectively, these results demonstrate a clear separation between pathogenic *Bacillus* species and *B. velezensis*, supporting the non-pathogenic status of strain D-18 at the species and strain levels.

**Table 1:** PathogenFinder-based prediction of human pathogenicity in representative pathogenic and non-pathogenic *Bacillus* species and *Lactiplantibacillus plantarum.*

Genus	Species	Strain	NCBI RefSeq assembly	PathogenFinder probability score	Matched non-pathogenic families	Matched pathogenic families
*Bacillus*	*anthracis*	Ames Ancestor A2084	GCF_000008445.1	**0.87**	13	**768**
	*cereus*	ATCC 10876	GCF_000160895.1	**0.824**	7	**63**
	*cereus*	ATCC 14579 P1	GCF_046524075.1	**0.874**	2	**256**
	*thuringiensis*	Serovar berliner ATCC 10792	GCF_000161615.1	**0.831**	6	**56**
	*thuringiensis*	BT407	GCF_000306745.1	**0.852**	4	**54**
	*licheniformis*	ATCC 14580	GCF_034478925.1	**0.808**	0	**441**
	*amyloliquefaciens*	GKT04	GCF_019396925.1	0.234	**119**	0
	*pumilus*	SAFR-032	GCF_000017885.4	0.231	**339**	0
	*subtilis*	168	GCF_000009045.1	0.24	**466**	0
*Lactiplantibacillus*	*plantarum*	SRCM100442	GCF_009913655.1	**0.184**	**13**	0

Bold values indicate representative extremes of predicted pathogenicity and matched pathogenic/non-pathogenic protein families within the dataset.

**Table 2:** PathogenFinder analysis of *Bacillus velezensis* D-18 and publicly available *B. velezensis* reference genomes.

Species	Strain	NCBI RefSeq assembly	PathogenFinder probability score	Matched non-pathogenic families	Matched pathogenic families
*Bacillus velezensis*	D-18	–	0.227	58	0
	CBMB205	GCF_002117165.1	0.224	77	0
	L-H15	GCF_000833005.1	0.227	56	0
	L-S60	GCF_000973485.1	0.227	56	0
	KACC 13105	GCF_000960265.2	0.224	77	0
	NKYL29	GCF_000740715.1	0.224	74	0
	AP183	GCF_000875875.1	0.232	118	0
	JJ-D34	GCF_000987825.1	0.224	56	0
	YJ11-1-4	GCF_000988345.1	0.231	80	0
	G341	GCF_001023595.1	0.231	102	0
	KCTC 13012	GCF_001267695.1	0.225	80	0
	NBIF-003	GCF_001440465.1	0.224	77	0
	NRRL B-41580	GCF_001461825.1	0.225	80	0
	OB9	GCF_001266815.1	0.228	108	0
	B26	GCF_001266825.1	0.228	108	0
	KACC 18228	GCF_001461835.1	0.231	96	0
	NRRL B-4257	GCF_001461845.1	0.225	79	0
	FKM10	GCF_001469675.1	0.224	77	0
	JS25R	GCF_000769555.1	0.225	72	0
	AS43.3	GCF_000319475.1	0.234	117	0
	FZB42	GCF_000015785.1	0.236	443	0

To further evaluate biosafety, acquired antimicrobial resistance (AMR) genes were screened using ResFinder v4.1 across an expanded panel of 89 antibiotics (Table 3). No acquired AMR genes were detected in the genome of *B. velezensis* D-18, indicating the absence of horizontally acquired resistance determinants. Similarly, other probiotic reference strains were largely devoid of acquired AMR genes. An exception was observed for *B. subtilis* 168, which showed predicted resistance to five antibiotics (streptomycin, spiramycin, telithromycin, tetracycline, and doxycycline) consistent with previously described intrinsic or strain-specific resistance traits rather than recent horizontal gene acquisition. In contrast, pathogenic *Bacillus* species exhibited a higher prevalence and broader spectrum of predicted resistance determinants. *B. cereus* ATCC 10876 displayed the most extensive resistance profile, with predicted resistance to seven antibiotics spanning multiple classes, including aminoglycosides (neomycin), β-lactams (amoxicillin and ampicillin), cephalosporins (ceftriaxone), tetracyclines (tetracycline and doxycycline), and polymyxins (colistin). Other pathogenic strains showed more limited resistance profiles, with resistance predictions largely restricted to fosfomycin. Overall, the absence of acquired AMR genes in *B. velezensis* D-18, together with the restricted and class-specific resistance patterns observed in pathogenic species, supports the genomic safety of strain D-18 with respect to antimicrobial resistance and its suitability as a probiotic candidate.

**Table 3:** Predicted antimicrobial resistance profiles based on genomic screening of *B. velezensis* D-18 and representative probiotic and pathogenic *Bacillus* species.

	Probiotic or pathogenic *Bacillus* spp. and *Lactiplantibacillus plantarum* test										
Antibiotic class	*B. velezensis D-18*	*B. subtilis 168*	*B. amyloliquefaciens GKT04*	*B. anthracis Ames Ancestor A2084*	*B. cereus* *ATCC 10876*	*B. cereus* *ATCC 14579 P1*	*B. licheniformis ATCC 14580*	*B. pumilus SAFR-032*	*B. thuringiensis serovar berliner ATCC 10792*	*B. thuringiensis Bt407*	*Lactiplantibacillus plantarum*
Gentamicin	**S**	S	**S**	S	S	S	**S**	S	S	S	**S**
Tobramycin	**S**	S	**S**	S	S	S	**S**	S	S	S	**S**
Streptomycin	**S**	**R**	**S**	S	S	S	**S**	S	S	S	**S**
Amikacin	**S**	S	**S**	S	S	S	**S**	S	S	S	**S**
Isepamicin	**S**	S	**S**	S	S	S	**S**	S	S	S	**S**
Dibekacin	**S**	S	**S**	S	S	S	**S**	S	S	S	**S**
Kanamycin	**S**	S	**S**	S	S	S	**S**	S	S	S	**S**
Neomycin	**S**	S	**S**	S	S	S	**S**	S	S	S	**S**
Lividomycin	**S**	S	**S**	S	S	S	**S**	S	S	S	**S**
Paromomycin	**S**	S	**S**	S	S	S	**S**	S	S	S	**S**
Ribostamycin	**S**	S	**S**	S	S	S	**S**	S	S	S	**S**
Unknown aminoglycoside	**S**	S	**S**	S	S	S	**S**	S	S	S	**S**
Butiromycin	**S**	S	**S**	S	S	S	**S**	S	S	S	**S**
Butirosin	**S**	S	**S**	S	S	S	**S**	S	S	S	**S**
Hygromycin	**S**	S	**S**	S	S	S	**S**	S	S	S	**S**
Netilmicin	**S**	S	**S**	S	S	S	**S**	S	S	S	**S**
Apramycin	**S**	S	**S**	S	S	S	**S**	S	S	S	**S**
Sisomicin	**S**	S	**S**	S	S	S	**S**	S	S	S	**S**
Arbekacin	**S**	S	**S**	S	S	S	**S**	S	S	S	**S**
Kasugamycin	**S**	S	**S**	S	S	S	**S**	S	S	S	**S**
Astromicin	**S**	S	**S**	S	S	S	**S**	S	S	S	**S**
Fortimicin	**S**	S	**S**	S	S	S	**S**	S	S	S	**S**
Spectinomycin	**S**	S	**S**	S	S	S	**S**	S	S	S	**S**
Fluoroquinolone	**S**	S	**S**	S	S	S	**S**	S	S	S	**S**
Ciprofloxacin	**S**	S	**S**	S	S	S	**S**	S	S	S	**S**
Unknown quinolone	**S**	S	**S**	S	S	S	**S**	S	S	S	**S**
Nalidixic acid	**S**	S	**S**	S	S	S	**S**	S	S	S	**S**
Amoxicillin	**S**	S	**S**	S	**R**	S	**S**	S	S	S	**S**
Amoxicillin+clavulanic acid	**S**	S	**S**	S	S	S	**S**	S	S	S	**S**
Ampicillin	**S**	S	**S**	S	**R**	S	**S**	S	S	S	**S**
Ampicillin+clavulanic acid	**S**	S	**S**	S	S	S	**S**	S	S	S	**S**
Cefepime	**S**	S	**S**	S	S	S	**S**	S	S	S	**S**
Cefixime	**S**	S	**S**	S	S	S	**S**	S	S	S	**S**
Cefotaxime	**S**	S	**S**	S	S	S	**S**	S	S	S	**S**
Cefoxitin	**S**	S	**S**	S	S	S	**S**	S	S	S	**S**
Ceftazidime	**S**	S	**S**	S	S	S	**S**	S	S	S	**S**
Ertapenem	**S**	S	**S**	S	S	S	**S**	S	S	S	**S**
Imipenem	**S**	S	**S**	S	S	S	**S**	S	S	S	**S**
Meropenem	**S**	S	**S**	S	S	S	**S**	S	S	S	**S**
Piperacillin	**S**	S	**S**	S	**R**	S	**S**	S	S	S	**S**
Piperacillin+tazobactam	**S**	S	**S**	S	S	S	**S**	S	S	S	**S**
Unknown beta-lactam	**S**	S	**S**	S	S	S	**S**	S	S	S	**S**
Aztreonam	**S**	S	**S**	S	S	S	**S**	S	S	S	**S**
Cefotaxime+clavulanic acid	**S**	S	**S**	S	S	S	**S**	S	S	S	**S**
Temocillin	**S**	S	**S**	S	S	S	**S**	S	S	S	**S**
Ticarcillin	**S**	S	**S**	S	S	S	**S**	S	S	S	**S**
Ceftazidime+avibactam	**S**	S	**S**	S	S	S	**S**	S	S	S	**S**
Penicillin	**S**	S	**S**	S	**R**	S	**S**	S	S	S	**S**
Ceftriaxone	**S**	S	**S**	S	S	S	**S**	S	S	S	**S**
Ticarcillin+clavulanic acid	**S**	S	**S**	S	S	S	**S**	S	S	S	**S**
Cephalothin	**S**	S	**S**	S	S	S	**S**	S	S	S	**S**
Cephalotin	**S**	S	**S**	S	S	S	**S**	S	S	S	**S**
Piperacillin+clavulanic acid	**S**	S	**S**	S	S	S	**S**	S	S	S	**S**
Ceftiofur	**S**	S	**S**	S	S	S	**S**	S	S	S	**S**
Sulfamethoxazole	**S**	S	**S**	S	S	S	**S**	S	S	S	**S**
Trimethoprim	**S**	S	**S**	S	S	S	**S**	S	S	S	**S**
Fosfomycin	**S**	S	**S**	**R**	**R**	**R**	**S**	S	**R**	**R**	**S**
Vancomycin	**S**	S	**S**	S	S	S	**S**	S	S	S	**S**
Teicoplanin	**S**	S	**S**	S	S	S	**S**	S	S	S	**S**
Bleomycin	**S**	S	**S**	S	S	S	**S**	S	S	S	**S**
Lincomycin	**S**	S	**S**	S	S	S	**S**	S	S	S	**S**
Clindamycin	**S**	S	**S**	S	S	S	**S**	S	S	S	**S**
Dalfopristin	**S**	S	**S**	S	S	S	**S**	S	S	S	**S**
Pristinamycin iia	**S**	S	**S**	S	S	S	**S**	S	S	S	**S**
Virginiamycin m	**S**	S	**S**	S	S	S	**S**	S	S	S	**S**
Quinupristin+dalfopristin	**S**	S	**S**	S	S	S	**S**	S	S	S	**S**
Tiamulin	**S**	S	**S**	S	S	S	**S**	S	S	S	**S**
Carbomycin	**S**	S	**S**	S	S	S	**S**	S	S	S	**S**
Erythromycin	**S**	S	**S**	S	S	S	**S**	S	S	S	**S**
Azithromycin	**S**	S	**S**	S	S	S	**S**	S	S	S	**S**
Oleandomycin	**S**	S	**S**	S	S	S	**S**	S	S	S	**S**
Spiramycin	**S**	**R**	**S**	S	S	S	**S**	S	S	S	**S**
Tylosin	**S**	S	**S**	S	S	S	**S**	S	S	S	**S**
Telithromycin	**S**	**R**	**S**	S	S	S	**S**	S	S	S	**S**
Tetracycline	**S**	**R**	**S**	S	**R**	S	**S**	S	S	S	**S**
Doxycycline	**S**	**R**	**S**	S	**R**	S	**S**	S	S	S	**S**
Minocycline	**S**	S	**S**	S	S	S	**S**	S	S	S	**S**
Tigecycline	**S**	S	**S**	S	S	S	**S**	S	S	S	**S**
Quinupristin	**S**	S	**S**	S	S	S	**S**	S	S	S	**S**
Pristinamycin ia	**S**	S	**S**	S	S	S	**S**	S	S	S	**S**
Virginiamycin s	**S**	S	**S**	S	S	S	**S**	S	S	S	**S**
Linezolid	**S**	S	**S**	S	S	S	**S**	S	S	S	**S**
Chloramphenicol	**S**	S	**S**	S	S	S	**S**	**R**	S	S	**S**
Florfenicol	**S**	S	**S**	S	S	S	**S**	S	S	S	**S**
Colistin	**S**	S	**S**	S	S	S	**S**	S	S	S	**S**
Fusidic acid	**S**	S	**S**	S	S	S	**S**	S	S	S	**S**
Mupirocin	**S**	S	**S**	S	S	S	**S**	S	S	S	**S**
Rifampicin	**S**	S	**S**	S	S	S	**S**	S	S	S	**S**
Metronidazole	**S**	S	**S**	S	S	S	**S**	S	S	S	**S**

(**S**), susceptible; (**R**), resistant. Resistance predictions are based on genomic screening using ResFinder v4.1 with a minimum sequence identity threshold of 90% and a minimum coverage of 60%. No acquired antimicrobial resistance genes were detected in the genome of *B. velezensis* D-18.

### 3.4 Prediction of probiotic-associated genes and secondary metabolites in the genome of *Bacillus velezensis* D-18

Comparative genomic analysis using BLAST and functional annotation enabled the identification of multiple genes associated with probiotic-relevant traits in *B. velezensis* D-18 (Table 4). These genes encompass functions related to environmental stress tolerance, host interaction, antimicrobial activity, and biofilm formation. Genes associated with oxidative and environmental stress resistance included catalase (*katA*), superoxide dismutase (*sodA*), and osmoprotectant transport systems such as *opuCB*. Additional genes linked to intestinal persistence and carbohydrate utilization, including *celB*, *treC*, and *xylA*, suggest metabolic adaptability within host-associated environments. Several genes involved in biofilm formation and regulatory control were also identified, including the transcriptional regulator *sinR*, its antagonist *sinI*, and matrix-associated components such as *bslA*. Sporulation-associated genes, including *spo0A*, *spoIIE*, and *spoVAC*, further support the ecological resilience and persistence potential of strain D-18. The genome additionally encodes multiple BGCs associated with antimicrobial lipopeptide production, including *srfAA–srfAD* (surfactin), *fenA* (fengycin), and *ituA* (iturin), as well as genes related to bacteriocin production such as *bacE*. The presence of the quorum-quenching enzyme gene *ytnP* further supports the ability of strain D-18 to interfere with quorum sensing systems in competing bacteria. Secondary metabolite BGCs were predicted using AntiSMASH v7.0, revealing seven clusters distributed across the genome (Table 5).

**Table 4:** Functional genes associated with probiotic traits and ecological fitness in *Bacillus velezensis* D-18.

Functional category	Gene/protein	Putative function
Acid and general stress tolerance	10 kDa chaperonin (Cpn10)	Protein folding under stress
	33 kDa chaperonin (Cpn33)	Stress-related protein stabilization
	60 kDa chaperonin (GroEL/Cpn60)	Protein folding and stress protection
	ATP-dependent protease subunit ClpY	Protein quality control
	ATP-dependent protease subunit ClpQ	Degradation of damaged proteins
	Cold shock protein CspB	Cold stress adaptation
	Cold shock protein CspC	RNA stabilization under stress
	Universal stress protein TeaD	General stress response
Adhesion and biofilm initiation	EpsD	Putative glycosyltransferase (EPS synthesis)
	EpsM	Putative acetyltransferase (EPS modification)
	EpsL	Putative sugar transferase
	EpsN	PLP-dependent aminotransferase
	Sortase D (SrtD)	Cell-surface protein anchoring
	Flagellin (Fla)	Motility and adhesion
	Lipoprotein signal peptidase (Lsp)	Lipoprotein maturation
	ABC transporter glutamine-binding protein (GlnH)	Nutrient uptake, adhesion
	Elongation factor Tu (EF-Tu)	Moonlighting adhesion protein
	TapA	Amyloid fiber assembly
	BslA	Biofilm surface layer protein
Bile salt tolerance	OppA	Oligopeptide transport
	DnaK	Molecular chaperone
	Enolase (Eno)	Stress response and adhesion
	Choloylglycine hydrolase (Cgh)	Bile salt deconjugation
	Sortase (Srt)	Cell surface protein anchoring
Intestinal persistence and carbohydrate utilization	CelB	PTS cellobiose transporter IIC
	TreC	Trehalose-6-phosphate hydrolase
	XylA	Xylose isomerase
Osmotic stress tolerance	Aqp	Aquaporin (water transport)
	OpuCB	Glycine betaine/carnitine/choline transporter
Non-ribosomal antimicrobial compounds	ituA	Iturin biosynthesis
	fenA	Fengycin biosynthesis
	srfAC	Surfactin biosynthesis
Bacteriocins	bacE	Bacilicin biosynthesis
Lipopeptides	srfAC	Surfactin biosynthesis
Sporulation and persistence	spo0A	Master sporulation regulator
	spoIIE	Stage II sporulation protein
	spoVAC	Stage V sporulation protein
	abrB	Transition-state transcriptional regulator
	sinI	Antagonist of SinR
	sinR	Master regulator of biofilm formation
Oxidative stress resistance	katA	Catalase
	sodA	Superoxide dismutase
Quorum quenching (QQ)	ytnP	AHL-lactonase (quorum quenching)
	bslA	Biofilm inhibition
Immune modulation (host interaction)	srfAA–AD	Surfactin complex
	fenA	Fengycin
	ituA	Iturin
	buk	Butyrate kinase (SCFA production)
Anti-inflammatory potential	buk	Butyrate production
Biofilm formation and regulation	sinR	Biofilm repression
	tasA	Amyloid fiber formation
	sipW	Signal peptidase (TasA processing)
	yqxM	Biofilm matrix protein
	sinI/sir	Biofilm regulatory balance

**Table 5:** Secondary metabolite biosynthetic gene clusters identified in the *Bacillus velezensis* D-18 genome by antiSMASH analysis.

Region	Type	From	To	Most similar known cluster	Similarity (%)
3.1	NRPS, transAT-PKS, betalactone	59,100	196,942	Fengycin	100
11.1	Lanthipeptide-class-II	147,596	170,784	Mersacidin	100
11.2	Other	310,950	352,368	Bacilysin	100
11.3	NRP-metallophore, NRPS	881,505	933,241	Bacillibactin	100
14.1	transAT-PKS, NRPS, T3PKS	1	106,474	Bacillaene	100
14.2	transAT-PKS	372,509	460,721	Macrolactin H	100
16.1	NRPS	193,542	258,952	Surfactin	82

These included clusters encoding the lipopeptides surfactin and fengycin, the lantibiotic mersacidin, the dipeptide antibiotic bacilysin, the siderophore bacillibactin, and the polyketide antibiotics bacillaene and macrolactin H. Most clusters exhibited 100% similarity to reference BGCs, whereas the surfactin cluster showed 82% similarity, indicating moderate divergence relative to previously characterized strains. Collectively, the presence of these genes and BGCs highlights the capacity of *B. velezensis* D-18 to produce a diverse repertoire of antimicrobial and bioactive compounds, supporting its ecological competitiveness and probiotic potential in aquaculture systems.

Most of the probiotic-associated genes identified in the strain D-18, including those related to stress tolerance (*katA*, *sodA*, *opuCB*), biofilm regulation (*sinR*, *sinI*, *bslA*), and sporulation (*spo0A*, *spoIIE*, *spoVAC*), are consistent with the conserved functional repertoire reported across *B. velezensis* genomes, where genes associated with environmental resilience, biofilm formation, and secondary metabolism are broadly distributed and contribute to ecological fitness and probiotic potential (Fan et al., 2017; Rabbee et al., 2019; Keshmirshekan et al., 2024; Wang et al., 2025). Similarly, several of the detected biosynthetic gene clusters, including those encoding surfactin, fengycin, bacillibactin, bacillaene, and macrolactin H, are widely distributed among *B. velezensis* strains and are considered characteristic of the species. In contrast, the moderate divergence observed in the surfactin biosynthetic cluster (82% similarity) and the presence of quorum-quenching-associated elements such as *ytnP* suggest potential strain-level variation in biosynthetic and functional capacity. Collectively, these findings indicate that the probiotic relevance of strain D-18 is not driven by uniquely encoded determinants, but rather by the integration of a conserved functional repertoire with specific strain-level variations.

### 3.5 Molecular docking analysis of mucin interactions

Molecular docking simulations were performed to explore potential interactions between a selected *B. velezensis* D-18 protein and a representative mucin glycoprotein model. Protein structures predicted with AlphaFold2 were used for docking analysis with mucin receptor models obtained from the Protein Data Bank. Docking simulations performed with AutoDock Vina generated multiple predicted binding conformations for each protein–mucin pair. The predicted binding affinities of the best-scoring docking poses ranged from approximately −6.4 to −9.0 kcal·mol^−1^.

**Figure 6:**
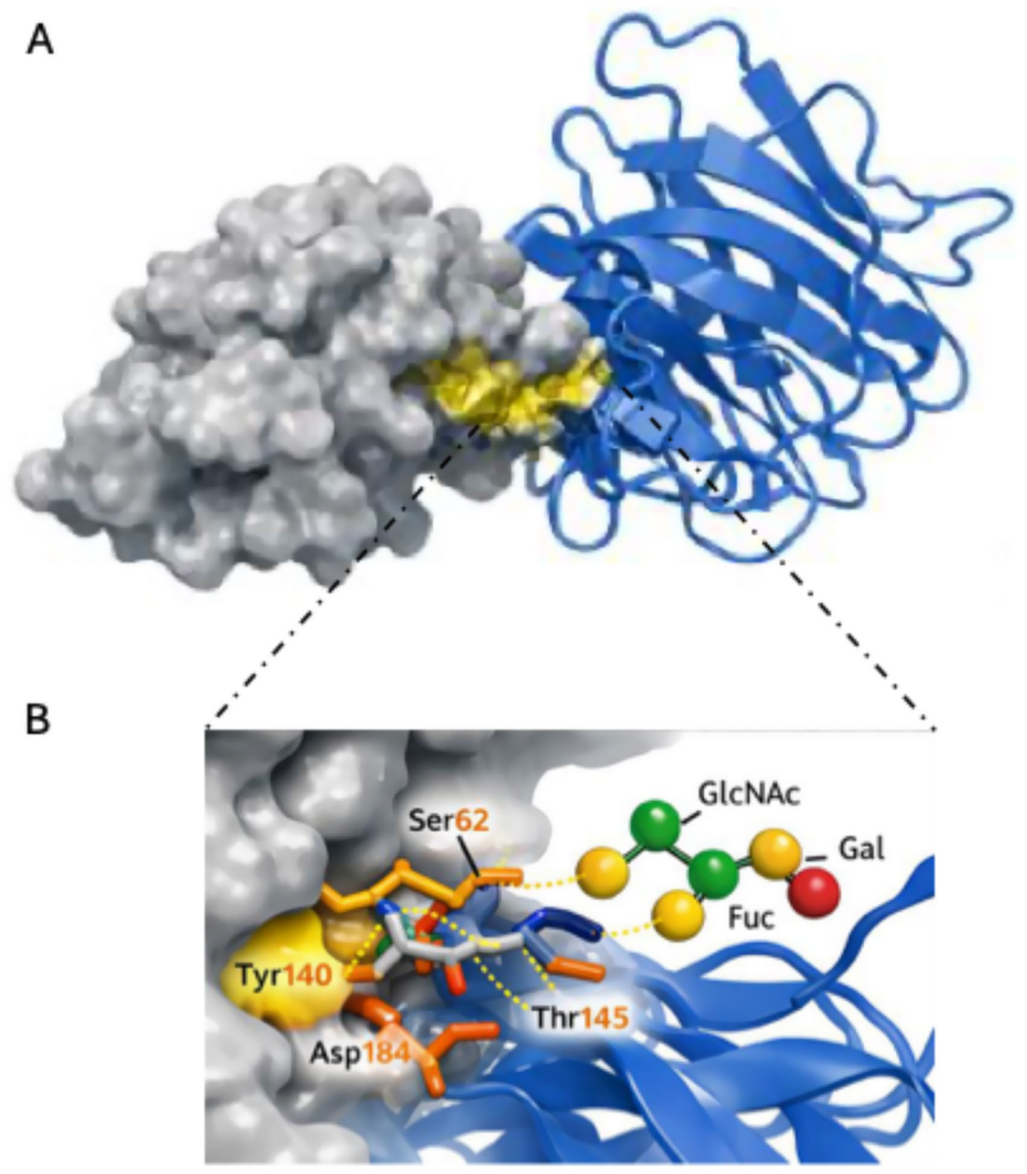
Representative molecular docking analysis of a *B. velezensis* D-18 protein with a mucin glycoprotein domain. **(A)** Predicted docking complex showing the interaction between a surface-associated protein of *B. velezensis* D-18 and a representative mucin glycoprotein domain. The bacterial protein is displayed as a surface model in white, while the mucin structure is represented as a blue-ribbon model. The predicted binding interface is highlighted. **(B)** Enlarged view of the docking interface illustrating potential hydrogen bonding and hydrophobic contacts between the bacterial protein and glycosylated regions of the mucin molecule. Hydrogen bonds are represented as dashed lines. Structural visualization was performed using PyMOL.

Structural inspection of the top-ranked docking poses revealed the formation of several hydrogen bonds and hydrophobic contacts between amino acid residues of the bacterial proteins and the mucin glycoprotein structures. The predicted complexes involved interactions with glycosylated regions of the mucin molecules. Representative docking conformations and interaction interfaces are shown in Figure 6. The predicted binding affinities of the best-scoring docking poses ranged from approximately −6.4 to 9.0 kcal·mol^−1^ across the evaluated protein-mucin pairs (Supplementary Table S2), which summarizes representative protein-mucin interactions selected according to predicted extracellular localization and functional relevance.

## 4 Discussion

The present study provides a comprehensive genome-based evaluation of *B. velezensis* D-18, integrating high-coverage genome sequencing, comparative genomics, safety assessment, and functional prediction analyses to support its inclusion as a probiotic for aquaculture applications. In our previous studies, we isolated and characterized this *B. velezensis* strain (Monzón-Atienza et al., 2021), provided evidence of the immunological mechanisms it confers to cultured fish, and demonstrated that it improves disease resistance in European seabass, including enhanced pathogen clearance and inhibition of *Vibrio* biofilm formation (Monzón-Atienza et al., 2022; Monzón-Atienza et al., 2024). However, the genomic determinants underlying these probiotic properties had not been systematically characterized.

The present work therefore connects experimentally observed probiotic phenotypes with their genomic basis. Genome sequencing revealed a draft genome of approximately 4.06 Mb containing 4,178 predicted protein-coding genes and 84 RNA genes. These values are consistent with previously reported genomes of *B. velezensis* within the *Bacillus subtilis* species complex (Fan et al., 2017; Rabbee et al., 2019). Functional annotation identified a broad distribution of genes across metabolic subsystems, with a predominance of genes associated with amino acid metabolism, carbohydrate utilization, and protein metabolism. Such metabolic versatility is characteristic of environmental *Bacillus* species and contributes to ecological adaptability in diverse environments, including aquaculture systems where nutrient availability and microbial competition fluctuate (Mingmongkolchai and Panbangred, 2018; Othoum et al., 2019).

Genome-informed analyses of probiotic *Bacillus* species have consistently demonstrated that key functional traits, including gastrointestinal stress tolerance, mucosal adhesion, and environmental resilience, are supported by conserved genetic determinants such as ATP synthase complexes, bile transport systems, and adhesion-related proteins. Comparative genomic studies in *Bacillus clausii* further indicate that these traits underpin probiotic persistence and host interaction (Ahire et al., 2021). More recent integrative genomic and phenotypic evidence further confirms that *Bacillus* strains capable of tolerating acidic pH and bile salts also harbor genetic determinants associated with adhesion, aggregation, and survival under gastrointestinal stress conditions, thereby reinforcing the predictive value of genome-based probiotic characterization (Söylemez-Milli, 2025).

Although inferences from the genome are striking, a growing body of evidence indicates that the functional relevance of probiotic candidates cannot be inferred solely from genomic annotation but requires integration with host-microbiome metabolic interactions and downstream physiological outcomes. Studies on lactic acid bacteria, particularly *Lactiplantibacillus plantarum*, have demonstrated that probiotic efficacy is frequently mediated through indirect modulation of the gut microbiota and the production of bioactive metabolites rather than direct colonization or single-gene effects. In Nile tilapia (*Oreochromis niloticus*), *L. plantarum* supplementation alleviates hepatic lipotoxicity by reshaping the intestinal microbial community and enriching microbial-derived tryptophan metabolites such as indole-3-propionic acid (IPA) and indole-3-acetic acid (IAA), which act systemically through activation of the aryl hydrocarbon receptor (Ahr) pathway to regulate immune and metabolic responses (Ding et al., 2025). Notably, these functional outcomes often emerge from community-level interactions rather than being directly encoded by the probiotic strain itself, highlighting the importance of microbiome-mediated mechanisms.

In this context, genome mining of *B. velezensis* D-18 reveals the presence of multiple genes associated with adhesion, stress tolerance, and secondary metabolite biosynthesis, which are consistent with classical probiotic selection criteria. However, similar to observations in *Lactobacillus* systems, these genomic features should be interpreted as indicators of ecological fitness and interaction potential rather than direct predictors of host benefit. The presence of biosynthetic gene clusters (e.g., surfactin, fengycin, bacillibactin) and mucin-interacting proteins suggests that D-18 may influence host physiology through modulation of microbial community structure, inhibition of opportunistic pathogens, and enhancement of mucosal barrier interactions. Importantly, the integration of these genomic traits with previously reported *in vivo* outcomes in European seabass, including improved survival following *V. anguillarum* challenge and enhanced mucosal adherence, supports a model in which *B. velezensis* D-18 exerts its probiotic effects through a combination of direct antimicrobial activity and indirect microbiome-mediated functional modulation.

Accurate taxonomic identification is essential for probiotic development, particularly within the *Bacillus* genus, which includes both beneficial and pathogenic species. The ANI-based analyses further provide important insights into the genomic positioning and safety profile of strain D-18. It clusters tightly with reference *B. velezensis* genomes, with ANI values consistently exceeding 97%, confirming its placement within a highly conserved species-level clade. In contrast, the extended comparison presented reveals a clear genomic distinction between D-18 and members of the *Bacillus cereus* group, including *B. anthracis*, *B. cereus*, and *B. thuringiensis*. Strain D-18 instead shows highest similarity to the *B. velezensis*–*B. amyloliquefaciens* group, a cluster widely associated with beneficial and probiotic traits. This separation is particularly relevant from a biosafety perspective, as it reinforces the absence of phylogenetic proximity to pathogenic *Bacillus* lineages and supports the classification of D-18 as a safe candidate for aquaculture applications (Fan et al., 2017; Keshmirshekan et al., 2024). This interpretation is consistent with PathogenFinder predictions, which classify *B. velezensis* strains as non-pathogenic.

The absence of any acquired antimicrobial resistance determinants in the genome of strain D-18 is particularly relevant in the context of probiotic safety. Previous studies have reported the presence of intrinsic or chromosomally encoded resistance genes in probiotic *Bacillus* strains, including macrolide resistance determinants such as *erm*-related genes in *Bacillus clausii* (Bozdogan et al., 2004). Although such resistance is often non-transferable, these findings underscore the importance of comprehensive genomic screening to exclude potentially mobilizable resistance elements in candidate probiotic strains. In the present study, genome-wide screening did not identify any acquired antimicrobial resistance genes in *B. velezensis* D-18, supporting its favorable safety profile. This is particularly important in light of increasing regulatory scrutiny regarding antimicrobial resistance dissemination through microbial feed additives, as current guidelines emphasize that probiotic candidates should lack transferable resistance genes and virulence determinants (EFSA, 2018; Kuebutornye et al., 2019). Collectively, these findings reinforce the suitability of *B. velezensis* D-18 for aquaculture applications.

Beyond safety considerations, the genome encodes multiple functional traits associated with probiotic activity. Among these, the presence of the quorum-quenching lactonase gene *ytnP* suggests the ability to interfere with acyl-homoserine lactone signaling pathways used by many Gram-negative pathogens, including *Vibrio* species. Disruption of quorum sensing is increasingly recognized as a promising strategy for attenuating bacterial virulence without imposing selective pressure associated with conventional antimicrobial agents (Subramani et al., 2025). Consistent with this prediction, previous work demonstrated that strain D-18 inhibits *Vibrio* biofilm formation through quorum sensing interference (Monzón-Atienza et al., 2024), and the genomic identification of *ytnP* provides a mechanistic explanation for this phenotype. While sequence variation was observed in the *ytnP* locus, the functional implications of these mutations remain to be experimentally validated and cannot be inferred solely from sequence-level analysis.

Genes associated with biofilm formation were also identified, including the conserved *yqxM–sipW–tasA* operon and its regulatory network involving *SinR* and *SlrA*. In *Bacillus* species, this regulatory system controls extracellular matrix production and biofilm architecture (Vlamakis et al., 2013). Biofilm formation can enhance microbial persistence in host-associated environments and facilitate competitive exclusion of opportunistic pathogens. In aquaculture settings characterized by complex microbial communities, the ability to establish stable biofilms may therefore contribute to probiotic persistence and ecological competitiveness.

In addition to their robust redox systems, probiotic bacteria also possess well-developed antioxidant enzymatic defenses comparable to those observed in higher organisms (Zolotukhin et al., 2018). Our genomic analysis revealed that *B. velezensis* D-18 encodes several genes associated with stress tolerance and environmental resilience, including catalase (*katA*), superoxide dismutase (*sodA*), and osmoprotectant transport systems. These genetic determinants likely contribute to the ability of the strain to withstand oxidative stress and fluctuating environmental conditions commonly encountered in aquatic environments and host-associated niches. Supporting this interpretation, bacteria have evolved multiple enzymatic mechanisms to control reactive oxygen species, including both Fe- and Mn-dependent superoxide dismutase as well as catalase activity, which together limit the formation of highly reactive hydroxyl radicals through the Fenton reaction (Wang et al., 2017). Moreover, the presence of sporulation-associated genes further enhances the ecological robustness of *Bacillus* probiotics, enabling persistence during feed processing, storage, and passage through the gastrointestinal tract (Bernardeau et al., 2017).

A notable feature of *B. velezensis* genomes is their extensive repertoire of secondary metabolite biosynthetic pathways. AntiSMASH analysis identified seven BGCs in strain D-18, including clusters encoding surfactin, fengycin, mersacidin, bacilysin, bacillibactin, bacillaene, and macrolactin H. These metabolites represent diverse classes of bioactive compounds with antimicrobial activity. Lipopeptides such as surfactin and fengycin are well known for their ability to disrupt microbial membranes and inhibit pathogen colonization (Tunsagool et al., 2021), while siderophores such as bacillibactin facilitate iron acquisition under nutrient-limited conditions. The coexistence of multiple BGCs suggests that strain D-18 possesses a broad chemical arsenal capable of suppressing competing microorganisms and shaping microbial community structure.

Although the genomic features identified in strain D-18 largely reflect the conserved functional repertoire of *B. velezensis*, several elements position this strain within the spectrum of intra-species variation. In particular, the moderate divergence observed in the surfactin biosynthetic cluster (82% similarity to reference clusters) suggests potential variation in lipopeptide structure or regulation, which may influence antimicrobial activity (Rabbee et al., 2019; Larsson and Flach, 2021). In addition, the presence and genomic context of the quorum-quenching gene *ytnP*, together with sequence variation identified within its coding and adjacent regions, may contribute to strain-level differences in quorum sensing interference capacity. More broadly, the integration of conserved traits, including stress tolerance, biofilm formation, sporulation, and secondary metabolism, with our previous demonstrations of phenotypic properties in European seabass (Monzón-Atienza et al., 2021; Monzón-Atienza et al., 2022; Monzón-Atienza et al., 2024) indicates that strain D-18 represents a functionally coherent variant within the *B. velezensis* lineage, rather than an outlier defined by unique genetic determinants (Fan et al., 2017; Wang et al., 2025).

Finally, molecular docking analyses provided structural insights into potential interactions between bacterial surface proteins and host mucin glycoproteins. Computational docking approaches have increasingly been applied to explore intermolecular interactions and binding stability in probiotic systems. For example, docking analyses have been used to characterize interaction between bacteriocins produced by *Limosilactobacillus fermentum* LMEM22 and bacterial target proteins such as sortase A (SrtA) and exotoxin A (ExtA) (Halder and Mandal, 2025). In the case of *B. velezensis* D-18, the predicted docking conformations suggested energetically favorable interactions with glycosylated mucin domains, primarily mediated through hydrogen bonds and hydrophobic contacts. Although these *in silico* predictions require experimental validation, they are consistent with previous observations demonstrating strong adhesion of strain D-18 to European seabass mucus (Monzón-Atienza et al., 2021; Monzón-Atienza et al., 2022). Mucosal adhesion is widely recognized as a key functional trait of probiotic microorganisms, as it facilitates the colonization of epithelial surfaces and promotes persistence within host-associated microbial communities.

Beyond adhesion and persistence, spore-forming *Bacillus* spp. are increasingly recognized as promising platforms for antigen delivery and mucosal immunomodulation in aquaculture. In particular, *Bacillus* spores have been explored as vaccine carriers capable of presenting heterologous antigens and stimulating both innate and adaptive immune responses at mucosal surfaces. Recent work has demonstrated that spore-based *Bacillus* formulations can modulate immune pathways in fish in a strain-dependent manner, highlighting their dual functionality as probiotics and immunological delivery systems (Gonçalves et al., 2026). In this context, the combined properties of environmental resilience, mucosal interaction, and immunomodulatory potential further reinforce the applicability of *B. velezensis* D-18 as a multifunctional biological tool for disease prevention in aquaculture.

Collectively, the integrative genomic analyses presented here provide a comprehensive framework linking the genomic architecture of *B. velezensis* D-18 to its previously demonstrated probiotic performance in European seabass. The absence of acquired antimicrobial resistance determinants and virulence-associated genes, together with the presence of diverse biosynthetic gene clusters encoding antimicrobial and bioactive metabolites, supports the biosafety and functional robustness of this strain. In addition, the identification of genes associated with quorum-quenching activity, stress tolerance, biofilm formation, and mucosal adhesion highlights multiple complementary mechanisms through which *B. velezensis* D-18 may contribute to pathogen suppression and host-microbe homeostasis in aquaculture environments. Importantly, these genomic features provide mechanistic support for earlier experimental observations showing enhanced host survival and inhibition of *Vibrio* biofilm formation. Taken together, our findings position *B. velezensis* D-18 as a genomically validated probiotic candidate and underscore the value of genome-resolved analyses for the rational selection and safety assessment of probiotic strains intended for sustainable aquaculture.

## Data Availability

The raw data generated in this study are available in the NCBI (https://www.ncbi.nlm.nih.gov) under accession number PRJNA1040484.

## Footnotes

1
https://proksee.ca

2
https://jspecies.ribohost.com/jspeciesws/#analyse

3
https://biit.cs.ut.ee/clustvis/

4
http://www.genomicepidemiology.org/services/

5
http://genepi.food.dtu.dk/resfinder

6
https://cge.food.dtu.dk/services/PathogenFinder/

7
https://antismash.secondarymetabolites.org/#!/start

8
https://autodock.scripps.edu

## References

[ref1] Acosta F. Izquierdo M. Montero D. Galindo-Villegas J. (2021). High-level biocidal products effectively eradicate pathogenic γ-Proteobacteria biofilms from aquaculture facilities. Aquaculture 532:766004. doi: 10.1016/j.aquaculture.2020.736004, 39175494 PMC11338163

[ref2] Ahire J. J. Kashikar M. S. Madempudi R. S. (2021). Comparative accounts of probiotic properties of spore and vegetative cells of *Bacillus clausii* UBBC07 and *in silico* analysis of probiotic function. 3 Biotech 11:116. doi: 10.1007/s13205-021-02668-0 PMC7867671 33604232

[ref3] Amit Pandey A. Tyagi A. Khairnar S. O. (2022). Oral feed-based administration of *Lactobacillus plantarum* enhances growth, haematological and immunological responses in *Cyprinus carpio*. Emerg. Anim. Species 3:100003. doi: 10.1016/J.EAS.2022.100003

[ref4] Aziz R. K. Bartels D. Best A. DeJongh M. Disz T. Edwards R. A. . (2008). The RAST server: rapid annotations using subsystems technology. BMC Genomics 9, 1–15. doi: 10.1186/1471-2164-9-75 18261238 PMC2265698

[ref5] Aziz T. Naveed M. Shabbir M. A. Sarwar A. Naseeb J. Zhao L. . (2024). Unveiling the whole genomic features and potential probiotic characteristics of novel *Lactiplantibacillus plantarum* HMX2. Front. Microbiol. 15:1504625. doi: 10.3389/fmicb.2024.1504625, 39611087 PMC11602494

[ref6] Bernardeau M. Lehtinen M. J. Forssten S. D. Nurminen P. (2017). Importance of the gastrointestinal life cycle of *Bacillus* for probiotic functionality. J. Food Sci. Technol. 54, 2570–2584. doi: 10.1007/s13197-017-2688-3, 28740315 PMC5502041

[ref7] Blin K. Shaw S. Augustijn H. E. Reitz Z. L. Biermann F. Alanjary M. . (2023). AntiSMASH 7.0: new and improved predictions for detection, regulation, chemical structures and visualisation. Nucleic Acids Res. 51, W46–W50. doi: 10.1093/nar/gkad344, 37140036 PMC10320115

[ref8] Bolger A. M. Lohse M. Usadel B. (2014). Trimmomatic: a flexible trimmer for Illumina sequence data. Bioinformatics 30, 2114–2120. doi: 10.1093/bioinformatics/btu170, 24695404 PMC4103590

[ref9] Bortolaia V. Kaas R. S. Ruppe E. Roberts M. C. Schwarz S. Cattoir V. . (2020). ResFinder 4.0 for predictions of phenotypes from genotypes. J. Antimicrob. Chem. 75, 3491–3500. doi: 10.1093/JAC/DKAA345, 32780112 PMC7662176

[ref10] Bozdogan B. Galopin S. Leclercq R. (2004). Characterization of a new erm-related macrolide resistance gene present in probiotic strains of *Bacillus clausii*. Appl. Environ. Microbiol. 70, 280–284. doi: 10.1128/AEM.70.1.280-284.2004, 14711653 PMC321311

[ref11] Cingolani P. Platts A. Wang L. L. Coon M. Nguyen T. Wang L. . (2012). A program for annotating and predicting the effects of single nucleotide polymorphisms, SnpEff: SNPs in the genome of *Drosophila melanogaster* strain w1118; iso-2; iso-3. Fly 6, 80–92. doi: 10.4161/fly.19695, 22728672 PMC3679285

[ref12] Cosentino S. Voldby Larsen M. Møller Aarestrup F. Lund O. (2013). PathogenFinder - distinguishing friend from foe using bacterial whole genome sequence data. PLoS One 8:e77302. doi: 10.1371/journal.pone.0077302, 24204795 PMC3810466

[ref13] Cutting S. M. (2011). *Bacillus* probiotics. Food Microbiol. 28, 214–220. doi: 10.1016/j.fm.2010.03.007 21315976

[ref14] Ding F. F. Zhou N. N. Mao Y. J. Yang J. Limbu S. M. Galindo-Villegas J. . (2025). *Lactiplantibacillus plantarum* attenuate gossypol-induced hepatic lipotoxicity by altering intestinal microbiota for enriching microbial tryptophan metabolites in Nile tilapia (*Oreochromis niloticus*). Microbiome 13:180. doi: 10.1186/s40168-025-02172-0, 40759977 PMC12323027

[ref15] EFSA (2018). Guidance on the characterization of microorganisms used as feed additives or as production organisms. J. EFSA 16:5206. doi: 10.2903/j.efsa.2018.5206 PMC7009341 32625840

[ref16] Ehling-Schulz M. Lereclus D. Koehler T. M. (2019). The *Bacillus cereus* group: *Bacillus* species with pathogenic potential. Microbiol. Spec. 7. doi: 10.1128/microbiolspec.GPP3-0032-2018 PMC6530592 31111815

[ref17] Fan B. Blom J. Klenk H. P. Borriss R. (2017). *Bacillus amyloliquefaciens*, *Bacillus velezensis*, and *Bacillus siamensis* form an “operational group *B. amyloliquefaciens*” within the *B. subtilis* species complex. Front. Microbiol. 8:22. doi: 10.3389/fmicb.2017.00022 28163698 PMC5247444

[ref18] Florensa A. F. Kaas R. S. Clausen P. T. L. C. Aytan-Aktug D. Aarestrup F. M. (2022). ResFinder – an open online resource for identification of antimicrobial resistance genes in next-generation sequencing data and prediction of phenotypes from genotypes. Microb. Genom. 8:000748. doi: 10.1099/mgen.0.000748 35072601 PMC8914360

[ref19] Galindo-Villegas J. Mulero V. (2014). “Current knowledge on the development and functionality of immune responses in the European sea bass (*Dicentrarchus labrax*),” in Biology of European Sea Bass, eds. Sánchez-Vázquez F. Muñoz-Cueto J. A. (Boca Raton, FL: CRC Press), 133–166. Taylor and Francis Group

[ref20] Garvey S. M. Mah E. Blonquist T. M. Kaden V. N. Spears J. L. (2022). The probiotic *Bacillus subtilis* BS50 decreases gastrointestinal symptoms in healthy adults: a randomized, double-blind, placebo-controlled trial in healthy adults. Gut Microbes 14:2122668. doi: 10.1080/19490976.2022.2122668, 36269141 PMC9590435

[ref21] Ghosh T. (2025). Recent advances in the probiotic application of the *Bacillus* as a potential candidate in the sustainable development of aquaculture. Aquaculture 594:741432. doi: 10.1016/j.aquaculture.2024.741432

[ref22] Gonçalves G. Santos R. A. Cerezo I. M. Gabriel T. Dias J. Magalhães R. . (2026). Antigen-displaying probiotic *Bacillus subtilis* spores induce subtle, strain-dependent immunomodulation in fish. Front. Mar. Sci. 13:1774354. doi: 10.3389/fmars.2026.1774354

[ref23] Halder D. Mandal S. (2025). The *in vitro* studies combined with molecular docking and MM-GBSA binding free energy calculations reveal broad-spectrum antibacterial activity of bacteriocin from curd-derived *Limosilactobacillus fermentum* with probiotic attributes. In silico Res. Biomed. 1:100009. doi: 10.1016/j.insi.2025.100009

[ref24] Keshmirshekan A. de Souza Mesquita L. M. Ventura S. P. M. (2024). Biocontrol manufacturing and agricultural applications of *Bacillus velezensis*. Trends Biotechnol. 42, 986–1001. doi: 10.1016/j.tibtech.2024.02.003, 38448350

[ref25] Kuebutornye F. K. A. Abarike E. D. Lu Y. (2019). A review on the application of *Bacillus* as probiotics in aquaculture. Fish Shellfish Immunol. 87, 820–828. doi: 10.1016/j.fsi.2019.02.010, 30779995

[ref26] Kumar V. Kumar Das B. Roy S. Bhowal P. Roy A. Kumar Jana A. . (2026). Exploring the host-pathogen interaction and genome analysis of multidrug-resistant bacterial pathogen *Proteus penneri* isolated from *Labeo rohita*. Front. Immunol. 17:1783987. doi: 10.3389/fimmu.2026.1733414 PMC13021476 41909648

[ref27] Larsson D. G. J. Flach C. F. (2021). Antibiotic resistance in the environment. Nat. Rev. Microbiol. 20, 257–269. doi: 10.1038/s41579-021-00649-x, 34737424 PMC8567979

[ref28] Li H. Durbin R. (2010). Fast and accurate long-read alignment with burrows–wheeler transform. Bioinformatics 26, 589–595. doi: 10.1093/bioinformatics/btp698 20080505 PMC2828108

[ref29] Li L. Wang Y. Li Y. Wang T. Wang Z. Zhou N. . (2026). Integrated microbiota and metabolomic analysis demonstrate the growth-promoting effects of *Lactobacillus plantarum* in Nile tilapia (*Oreochromis nilotica*). Anim. Microbiome 8:14. doi: 10.1186/s42523-026-00516-1, 41535999 PMC12888601

[ref30] Metsalu T. Vilo J. (2015). ClustVis: a web tool for visualizing clustering of multivariate data using principal component analysis and heatmap. Nuclic Acids Res. 43, W566–W570. doi: 10.1093/nar/gkv468, 25969447 PMC4489295

[ref31] Mingmongkolchai S. Panbangred W. (2018). Bacillus probiotics: an alternative to antibiotics for livestock production. J. Appl. Microbiol. 124, 1334–1346. doi: 10.1111/JAM.13690 29316021

[ref32] Mitropoulou A. Mougiakou E. Koumparelou A. Samaras A. Seriatos A. Pavlidis M. (2026). Developing operational welfare indicators for European Sea bass (*Dicentrarchus labrax*): Delphi-informed reflections on advancing farmed fish welfare. Aquaculture 614:743474. doi: 10.1016/j.aquaculture.2025.743474

[ref34] Montalban-Arques A. De Schryver P. Bossier P. Gorkiewicz G. Mulero V. Gatlin D. M. . (2015). Selective manipulation of the gut microbiota improves immune status in vertebrates. Front. Immunol. 6:512. doi: 10.3389/fimmu.2015.00512, 26500650 PMC4598590

[ref35] Monzón-Atienza L. Bravo J. Fernández-Montero Á. Charlie-Silva I. Montero D. Ramos-Vivas J. . (2022). Dietary supplementation of *Bacillus velezensis* improves *Vibrio anguillarum* clearance in European sea bass by activating essential innate immune mechanisms. Fish Shellfish Immunol. 124, 244–253. doi: 10.1016/J.FSI.2022.03.032, 35421573

[ref36] Monzón-Atienza L. Bravo J. Torrecillas S. Gómez-Mercader A. Montero D. Ramos-Vivas J. . (2024). An in-depth study on the inhibition of quorum sensing by *Bacillus velezensis* D-18: its significant impact on *Vibrio* biofilm formation in aquaculture. Microorganisms 12:890. doi: 10.3390/microorganisms12050890, 38792721 PMC11123725

[ref37] Monzón-Atienza L. Bravo J. Torrecillas S. Montero D., Canales, A. F. G. de, de la Banda I. G. Galindo-Villegas J. Ramos-Vivas J. Acosta F. (2021). Isolation and characterization of a *Bacillus velezensis* D-18 strain, as a potential probiotic in European seabass aquaculture. Prob. Antimicrob. Prot., 13, 1404–1412. doi: 10.1007/s12602-021-09782-8, 33811608

[ref39] Nayak S. K. (2020). Multifaceted applications of probiotic *Bacillus* species in aquaculture with special reference to *Bacillus subtilis*. Rev. Aqua. 13, 862–906. doi: 10.1111/raq.12503

[ref40] Othoum G. Prigent S. Derouiche A. Shi L. Bokhari A. Alamoudi S. . (2019). Comparative genomics study reveals Red Sea *Bacillus* with characteristics associated with potential microbial cell factories (MCFs). Sci. Rep. 9:19254. doi: 10.1038/s41598-019-55726-2, 31848398 PMC6917714

[ref42] Rabbee M. F. Sarafat Ali M. Choi J. Hwang B. S. Jeong S. C. Baek K. H. (2019). *Bacillus velezensis*: a valuable member of bioactive molecules within plant microbiomes. Molecules 24:1046. doi: 10.3390/molecules24061046, 30884857 PMC6470737

[ref43] Rahayu S. Amoah K. Huang Y. Cai J. Wang B. Shija V. M. . (2024). Probiotics application in aquaculture: its potential effects, current status in China and future prospects. Front. Mar. Sci. 11:1455905. doi: 10.3389/fmars.2024.1455905

[ref44] Ramirez-Olea H. Reyes-Ballesteros B. Chavez-Santoscoy R. A. (2022). Potential application of the probiotic *Bacillus licheniformis* as an adjuvant in the treatment of diseases in humans and animals: a systematic review. Front. Microbiol. 13:993451. doi: 10.3389/fmicb.2022.993451, 36225361 PMC9549136

[ref45] Richter M. Rosselló-Móra R. Oliver Glöckner F. Peplies J. (2016). JSpeciesWS: a web server for prokaryotic species circumscription based on pairwise genome comparison. Bioinformatics 32, 929–931. doi: 10.1093/bioinformatics/btv681, 26576653 PMC5939971

[ref47] Schar D. Klein E. Y. Laxminarayan R. Gilbert M. Van Boeckel T. (2020). Global trends in antimicrobial use in aquaculture. Sci. Rep. 10:21878. doi: 10.1038/s41598-020-78849-3, 33318576 PMC7736322

[ref48] Shija V. M. Chen H. Li Y. Ng’onga L. Amoah K. Yong Z. . (2025). Effects of dietary supplementation with fish-derived *Bacillus amyloliquefaciens* AV5 on growth status, immune response, microbiota, and intestinal health of Nile tilapia (*Oreochromis niloticus*). Aqua. Rep. 41:102658. doi: 10.1016/j.aqrep.2025.102658

[ref49] Söylemez-Milli N. (2025). The resistance abilities of some Bacillus species to gastrointestinal tract conditions: whole geneome sequencing of the novel candidate probiotic strains *Bacillus clausii* BA8 and *Bacillus subtilis* BA11. Food Sci. Nut. 13:e70018. doi: 10.1002/fsn3.70018, 39911839 PMC11795423

[ref50] Subramani T. Saravanan H. David H. Solanke J. Rajaramon S. Dandela R. . (2025). Bioorganic compounds in quorum sensing disruption: strategies, mechanisms, and future prospects. Bioorg. Chem. 156:108192. doi: 10.1016/j.bioorg.2025.108192, 39874908

[ref51] Tao L. Lu H. Xiong J. Zhang L. Sun W. W. Shan X. F. (2024). The application and potential of postbiotics as sustainable feed additives in aquaculture. Aquaculture 592:741237. doi: 10.1016/j.aquaculture.2024.741237

[ref52] Tarasov A. Vilella A. J. Cuppen E. Nijman I. J. Prins P. (2015). Sambamba: fast processing of NGS alignment formats. Bioinformatics 31, 2032–2034. doi: 10.1093/bioinformatics/btv098, 25697820 PMC4765878

[ref53] Torres M. Sampedro I. Llamas I. Béjar V. (2026). Bacillus velezensis. Trends Microbiol. 34, 113–114. doi: 10.1016/j.tim.2025.07.010 40883211

[ref54] Trott O. Olson J. (2010). AutoDock Vina: improving the speed and accuracy of docking with a new scoring function, efficient optimization and multithreading. J. Comp. Chem. 31, 455–461. doi: 10.1002/jcc.21334, 19499576 PMC3041641

[ref55] Tunsagool P. Ploypetch S. Jaresitthikunchai J. Roytrakul S. Choowongkomon K. Rattanasrisomporn J. (2021). Eficacy of cyclic lipopeptides obtained from *Bacillus subtilis* to inhibit the growth of *Microsporum canis* isolated from cats. Heliyon 7:e07980. doi: 10.1016/j.heliyon.2021.e07980, 34585007 PMC8450251

[ref56] Vlamakis H. Chai Y. Beauregard P. Losick R. Kolter R. (2013). Sticking together: building a biofilm the *Bacillus subtilis* way. Nat. Rev. Microbiol. 11, 157–168. doi: 10.1038/nrmicro2960, 23353768 PMC3936787

[ref57] Wang Y. Wu Y. Wang Y. Xu H. Mei X. Yu D. . (2017). Atioxidant properties of probiotic bateria. Nutrients 9:521. doi: 10.3390/nu9050521 28534820 PMC5452251

[ref58] Wang Z. Xu T. Wang J. Sun G. Chen Q. Sun H. . (2025). Probiotic potential of *Bacillus velezensis* MZ-09: assessing characteristics and safety through in vitro and in vivo analyses. Microbiol. Res. 301:128321. doi: 10.1016/j.micres.2025.128321, 40845728

[ref59] Woldemariamyohannes K. Wan Z. Yu Q. Li H. Wei X. Liu Y. . (2020). Prebiotic, probiotic, antimicrobial, and functional food applications of *Bacillus amyloliquefaciens*. J. Agri. Food Chem. 68, 14709–14727. doi: 10.1021/acs.jafc.0c06396, 33280382

[ref60] Yu H. Nazir S. Ijaz F. Zahid M. U. Mushtaq M. Khan M. . (2025). Dietary supplementation of *Bacillus subtilis* as probiotic influenced the growth performance, hematological parameters, immune function, antioxidant status, and digestive enzyme activity of Nile Tilapia fingerlings (*Oreochromis niloticus*). Animals 15:1256. doi: 10.3390/ani15091256, 40362071 PMC12071117

[ref61] Zolotukhin P. V. Prazdnova E. V. Chistyakov V. A. (2018). Methods to assess the antioxidative properties of probiotics. Probiotics Antimicrob. Proteins 10, 589–599. doi: 10.1007/s12602-017-9375-6 29249065

